# Epidemiological Profile and Risk Factors of Stroke in a Tertiary Care Hospital: A Cross-Sectional Study

**DOI:** 10.7759/cureus.94241

**Published:** 2025-10-09

**Authors:** Nand Kishor Prasad Sah, Himani Rathi, Shipra Gangwar, Ranjit Tiwari, Dimpal Pal

**Affiliations:** 1 Physiotherapy, Teerthanker Mahaveer University, Moradabad, IND

**Keywords:** diabetes mellitus, hypertension, intracerebral haemorrhage, risk factors, stroke

## Abstract

Introduction: Stroke remains a most important public health apprehension in India, particularly in underserved regions. Understanding local stroke epidemiology and associated risk factors is essential for developing targeted interventions. This study aims to evaluate the distribution of stroke types, assess associated risk factors, and analyse clinical features and outcomes among stroke patients admitted to a tertiary care hospital.

Methods: This descriptive, cross-sectional study included 148 stroke patients admitted between January 2023 and December 2024. Data were collected retrospectively from medical records. Demographic information, stroke subtypes, risk factors, clinical features, and outcomes were analysed using IBM SPSS Statistics for Windows, Version 27 (Released 2020; IBM Corp., Armonk, New York). Chi-square tests, logistic regression, and descriptive statistics were applied.

Results: Intracerebral haemorrhage was the most common stroke type (85.1%), followed by epidural haemorrhage (8.8%), subdural haemorrhage (5.4%), and subarachnoid haemorrhage (0.7%). Hypertension (45.3%) and diabetes mellitus (50%) were the most prevalent risk factors; both were significantly associated with haemorrhagic stroke (p < 0.001). The mean age was 65.15 ± 7.87 years, and the average Glasgow Coma Scale (GCS) score at presentation was 9.49 ± 2.14. No significant gender-based difference in stroke subtype distribution was observed (P=0.412).

Conclusion: This study concluded that there is a predominance of intracerebral haemorrhage among hospitalised stroke patients in a tertiary care setting in Moradabad, with hypertension and diabetes mellitus emerging as the most significant and common modifiable risk factors.

## Introduction

Stroke is an important cause of death and long-term disability worldwide, posing an important problem for individuals, families, and healthcare systems. In India, stroke accounts for around 11% of all deaths and is the second most common cause of death after ischaemic heart disease [1]. It is estimated that nearly 13.7 million people suffer a stroke each year, with 5.5 million succumbing to its effects [2]. Low- and middle-income countries (LMICs) account for over 85% of global stroke-related deaths and more than 80% of the total disability-adjusted life years lost due to stroke [3].

India, as one of the most populous LMICs, has observed an important epidemiological transition with non-communicable diseases such as stroke becoming increasingly prevalent. The incidence of stroke in India has been rising, with an estimated annual incidence of 119-145 per 100,000 population and a prevalence of approximately 0.5-0.7% in rural and urban situations, respectively [4,5]. This rising problem is attributed to numerous factors, including increased life expectancy, urbanisation, changing lifestyle behaviours, and a flow of adjustable risk factors such as hypertension, diabetes mellitus, dyslipidaemia, smoking, and physical inactivity [6].

The types of strokes, mainly categorised as ischaemic and haemorrhagic, differ in aetiology, management, and prognosis. Ischaemic strokes account for approximately 80-85% of all strokes, primarily resulting from thromboembolic occlusion of cerebral vessels, whereas haemorrhagic strokes comprise about 15-20% and involve bleeding into the brain parenchyma or subarachnoid space [7]. Understanding the local distribution of these stroke types is crucial to developing region-specific diagnostic and treatment protocols.

Multiple studies from different Indian regions have identified variations in stroke epidemiology, risk factors, and clinical consequences. For example, urban populations show a higher incidence of ischaemic stroke, frequently associated with sedentary lifestyles and metabolic syndrome, while rural populations tend to have higher rates of haemorrhagic stroke due to poorly controlled hypertension and lack of healthcare access [8,9]. However, complete, region-specific data are still scarce, predominantly in states, the most populous state in India, which also bears a considerable burden of non-communicable diseases but remains under-represented in stroke investigations.

A major city presents a unique healthcare landscape, characterised by urban-rural overlap, sociocultural diversity, and varying levels of healthcare accessibility. In spite of the growing number of stroke admissions in tertiary care hospitals in this region, there remains limited data on stroke subtypes, risk factor profiles, clinical presentations, and patient outcomes. The present literature frequently provides generalised national or state-level identifications, which may not adequately reflect ground-level realities. Moreover, differences in risk factor occurrences, patient behaviour, and healthcare-seeking patterns across different Indian regions necessitate localised data to inform public health methods.

In this context, the current study intends to fill this serious knowledge gap by analysing stroke patterns, associated risk factors, clinical features, and results from patients admitted to a tertiary care hospital. The study utilises a descriptive cross-sectional design based on a retrospective review of medical records, focusing on patients diagnosed with stroke within three days of onset. By evaluating both ischaemic and haemorrhagic stroke cases and identifying important demographic and clinical variables, this investigation seeks to develop actionable understandings for clinicians, public health planners, and policymakers.

The expected outcomes of this study include a deeper understanding of the epidemiological profile of stroke, identification of high-risk groups, and the development of region-specific prevention and management methods. These results may also contribute to the broader national stroke database and facilitate future multicentric research. Therefore, this study focuses on improving stroke care delivery and its outcomes in underserved regions through evidence-based, local understandings. This study aimed to describe the epidemiological profile, stroke subtypes, and associated risk factors among stroke patients admitted to a tertiary care hospital in Moradabad.

## Materials and methods

Research design

This is a retrospective observational study. The study included patients who visited the hospital between January 2023 and December 2024, comprising 148 consecutive stroke patients. The study reviewed patient records and evaluated the outcome. The primary outcomes were stroke subtype distribution and in-hospital mortality. Exposures included hypertension, diabetes, dyslipidaemia, and other vascular risk factors. It was demonstrated at a tertiary care teaching hospital that caters to a large catchment area, including both urban and rural populations. All diagnoses were confirmed by neurologists using standard clinical and neuroimaging criteria to ensure diagnostic accuracy. Neuroimaging was performed in all cases using non-contrast CT as the first-line modality, with MRI performed where clinically indicated. Stroke severity was assessed using the National Institutes of Health Stroke Scale (NIHSS) at the time of admission. The study population comprised all patients diagnosed with stroke and admitted to the neurology and internal medicine wards during the specified period.

Inclusion criteria

The inclusion criteria for this study were as follows: patients with a confirmed diagnosis of haemorrhagic stroke only, based on clinical features and radiological imaging; admission to the hospital within three days of stroke symptom onset to ensure accurate documentation of clinical presentation and early outcomes; age ≥ 18 years; and complete medical records, including demographic details, clinical history, imaging findings, laboratory investigations, treatment details, and consequences.

Exclusion criteria

Exclusion criteria included incomplete or missing medical records, stroke cases admitted more than three days after symptom onset, as delayed presentations could influence outcomes and confound clinical data, recurrent stroke admissions within the study period (only the first episode was considered to avoid duplication), and transient ischaemic attacks, which do not represent completed strokes.

Data collection

A pre-designed structured data extraction form was active to systematically collect relevant information from patient records. To minimise selection bias, all consecutive eligible patients during the study period were included. Although the study was conducted at a single tertiary care hospital in Moradabad, this centre serves as a referral hub for both urban and rural populations in the region, making the study population reasonably representative. Nevertheless, the single-centre setting is acknowledged as a limitation, and multicentric studies in Moradabad would improve replicability. The study size reflects all eligible stroke admissions during the period; no formal sample size calculation was required. Variable quantity collected included demographic details such as age, sex, and residence; clinical characteristics such as presenting symptoms, Glasgow Coma Scale scores, blood pressure readings, history of seizures, and level of consciousness at presentation; stroke classification into ischaemic or haemorrhagic based on imaging results; and risk factors including hypertension, diabetes mellitus, dyslipidaemia, smoking, alcohol use, atrial fibrillation, previous history of stroke or transient ischaemic attack, and family history of stroke.

Haemorrhagic stroke subtypes were further categorised into intracerebral haemorrhage (ICH), subarachnoid haemorrhage (SAH), subdural haemorrhage (SDH), and extradural haematoma). Severity grading was extracted from NIHSS documentation. Additionally, laboratory and radiological parameters, including blood glucose levels, lipid profiles, and neuroimaging results, were noted. Treatment-related data, including the administration of thrombolytic therapy, antihypertensive agents, antiplatelet agents, and statins, were also documented. For haemorrhagic strokes, treatment followed standard hospital protocols, including blood pressure control, osmotic agents such as mannitol when indicated, seizure prophylaxis if clinically required, and supportive neurocritical care.

Outcome analysis

Outcomes assessed included discharge status, length of hospital stay, in-hospital mortality, and complications like aspiration pneumonia, sepsis, or deep vein thrombosis. All patient data were anonymised during extraction and analysis to maintain privacy and uphold ethical standards.

Statistical analysis

Data were entered in Microsoft Excel (Microsoft Corporation, Redmond, Washington) and analysed using IBM SPSS Statistics for Windows, Version 27 (Released 2020; IBM Corp., Armonk, New York). Descriptive statistics were used to summarise demographic and clinical variables, with categorical data presented as frequencies and percentages and continuous variables expressed as means ± standard deviations or medians with interquartile ranges. Associations between categorical variables were assessed using the chi-square test or Fisher's exact test, while the independent t-test or Mann-Whitney U test was used to compare continuous variable quantity based on data distribution. Univariate logistic regression was performed to identify risk factors associated with poor results, such as in-hospital mortality or prolonged hospitalisation. Multivariate logistic regression was used to adjust for the confounding variable quantity and to identify independent predictors of death and problems. Results were reported as odds ratios with 95% confidence intervals, and a p<0.05 was considered a significant difference.

## Results

Table 1 shows the descriptive statistics for 148 stroke patients, revealing a mean age of 65.15 ± 7.87 years, indicating that most patients were elderly, aligning with the known higher stroke risk in older populations. The systolic blood pressure ranged from 130 to 150 mmHg, with a mean of 141.52 ± 4.27 mmHg, suggesting that many patients were hypertensive at the time of admission, a major modifiable risk factor for stroke. The diastolic blood pressure had a mean value of 93.75 ± 5.4 mmHg, which is also elevated, reinforcing the prevalence of hypertension in the cohort. The Glasgow Coma Scale scores, used to assess the level of consciousness, ranged from 6 to 13, with a mean score of 9.49 ± 2.14, indicating that most patients presented with moderate impairment in consciousness, consistent with the severity of their acute stroke.

**Table 1:** Baseline characteristics of the patients SD = Standard Deviation; SBP = Systolic Blood Pressure; DBP = Diastolic Blood Pressure; GCS = Glasgow Coma Scale

Parameter	N	Minimum	Maximum	Mean±SD
Age	148	50.00	88.00	65.15±7.87
SBP	148	130.00	150.00	141.52±4.27
DPB	148	80.00	110.00	93.75±5.4
GCS	148	6.00	13.00	9.49±2.14

Out of 148 stroke patients, 82 were male and 66 were female. Among males, the most common stroke subtype was ICH (n=69), followed by epidural haemorrhage (EDH) (n=9), subdural haemorrhage (SDH) (n=3), and SAH (n=1). Similarly, among females, ICH remained the predominant subtype (n=57), with smaller numbers experiencing EDH (n=4) and SDH (n=5); no cases of SAH were reported in females. The chi-square test yielded a value of χ² = 2.870 with a p-value of 0.412, indicating that there is no statistically significant association between gender and type of stroke in this study population (Table 2).

**Table 2:** Types of strokes and gender with their analysis Chi-square; P<0.05 is significant. EDH = Epidural Haemorrhage; ICH = Intracerebral Haemorrhage; SAH = Subarachnoid Haemorrhage; SDH = Subdural Haemorrhage

Gender	Types of Strokes				Total	χ2	P-value*
	EDH	ICH	SAH	SDH			
Male	9	69	1	3	82	2.870	0.412
Female	4	57	0	5	66		

The present analysis explored the association between common risk factors and different types of strokes among 148 patients admitted to a tertiary care hospital. ICH emerged as the most prevalent stroke subtype, accounting for 126 cases (85.1%), followed by EDH with 13 cases, SDH with eight cases, and a single case of SAH. Among the various risk factors assessed, cardiovascular disease was observed in only one patient (0.7%), exclusively associated with the SAH case, a specific but rare association. Diabetes mellitus was present in 74 patients (50%), predominantly among those with ICH (82.4%), and to a lesser extent in patients with EDH (9.5%) and SDH (8.1%). Hypertension was recognised in 67 patients (45.3%), with 91% of those cases occurring in individuals with ICH, affirming its strong link to haemorrhagic stroke. A previous history of stroke was noted in five patients (3.4%), more frequently in ICH (60%) and SDH (40%) cases. Tobacco use was reported in one patient (0.7%), who had ICH. The chi-square test yielded a value of 165.042 with a p-value of <0.001, representing a highly significant association between the type of stroke and the presence of specific risk factors (Table 3).

**Table 3:** Correlation between the risk factors and stroke types *Bivariate correlation; **Highly significant. EDH = Epidural Haemorrhage; ICH = Intracerebral Haemorrhage; SAH = Subarachnoid Haemorrhage; SDH = Subdural Haemorrhage

Risk Factor and Other Parameters	Types of Strokes				Total	Value*	P-value**
	EDH	ICH	SAH	SDH			
Cardiovascular Disease	0	0	1	0	1	165.042	<0.001
% within RISK_FACTORS	0.00%	0.00%	100.00%	0.00%	100.00%		
% within TYPES_OF_STROKE	0.00%	0.00%	100.00%	0.00%	0.70%		
% of Total	0.00%	0.00%	0.70%	0.00%	0.70%		
Diabetes Mellitus	7	61	0	6	74		
% within RISK_FACTORS	9.50%	82.40%	0.00%	8.10%	100.00%		
% within TYPES_OF_STROKE	53.80%	48.40%	0.00%	75.00%	50.00%		
% of Total	4.70%	41.20%	0.00%	4.10%	50.00%		
Hypertension	6	61	0	0	67		
% within RISK_FACTORS	9.00%	91.00%	0.00%	0.00%	100.00%		
% within TYPES_OF_STROKE	46.20%	48.40%	0.00%	0.00%	45.30%		
% of Total	4.10%	41.20%	0.00%	0.00%	45.30%		
Previous Stroke	0	3	0	2	5		
% within RISK_FACTORS	0.00%	60.00%	0.00%	40.00%	100.00%		
% within TYPES_OF_STROKE	0.00%	2.40%	0.00%	25.00%	3.40%		
% of Total	0.00%	2.00%	0.00%	1.40%	3.40%		
Tobacco Usage	0	1	0	0	1		
% within RISK_FACTORS	0.00%	100.00%	0.00%	0.00%	100.00%		
% within TYPES_OF_STROKE	0.00%	0.80%	0.00%	0.00%	0.70%		
% of Total	0.00%	0.70%	0.00%	0.00%	0.70%		
Total	13	126	1	8	148		
% within RISK_FACTORS	8.80%	85.10%	0.70%	5.40%	100.00%		
% within TYPES_OF_STROKE	100.00%	100.00%	100.00%	100.00%	100.00%		
% of Total	8.80%	85.10%	0.70%	5.40%	100.00%		

## Discussion

This study demonstrated a predominance of ICH (85%) among stroke patients, with hypertension (45.3%) and diabetes (50%) as major associated risk factors (P<0.001). This descriptive study of 148 stroke patients admitted to a tertiary hospital revealed several noteworthy results, particularly the predominance of ICH, the high prevalence of hypertension and diabetes, and the significant associations between these risk factors and stroke subtypes. While haemorrhagic strokes constitute 10-20% of all strokes globally, our cohort established an unusually high proportion of ICH (85%) among haemorrhagic strokes. This aligns with previous Indian hospital-based studies reporting elevated haemorrhagic stroke rates, up to 30% in eastern regions, probably due to regional differences and referral biases for severe cases [10].

Hypertension emerged as the most prevalent risk factor (45%) and was strongly linked with ICH (91% of hypertensive patients). These results echo global evidence, including the INTERSTROKE case-control study, where hypertension conferred a doubled risk of haemorrhagic stroke, with population-attributable risks around 35%. A Korean prospective cohort study, which reinforces similar data, found that a 20 mmHg rise in systolic blood pressure increased the risk of ICH more than that of SAH [11]. In addition, older case-control and cohort data confirm hypertension as the leading modifiable contributor to ICH [12].

Diabetes mellitus, which is present in 50% of patients, often overlaps with hypertension, reinforcing the well-recognised clustering of metabolic risk factors among stroke sufferers in India. In our data, diabetes was more prevalent in ICH cases (82%) and absent in the single SAH. However, meta-analyses suggest diabetes modestly increases ICH risk (RR ~1.30) [13]; our higher prevalence likely reflects Indian-specific trends of increasing diabetes and poor control.

Comparative analysis with ischaemic stroke cohorts suggests haemorrhagic stroke risk relies more heavily on hypertension and alcohol use, while diabetes tends to have a stronger link to ischaemic types [14]. Such patterns were echoed in an Egyptian study, where diabetic patients exhibited higher ischaemic stroke rates (93% vs. 83%) and lower haemorrhagic stroke incidence, underscoring the essential role of diabetes in ischaemic pathophysiology. In contrast, our haemorrhagic cohort shows overlapping risk influences, necessitating nuanced local interpretation.

The near-absence of tobacco use and cardiovascular disease in our cohort may reflect underreporting or referral patterns, but these differences with global INTERSTROKE results, where smoking and cardiac conditions accounted for ~19% and ~7% of haemorrhagic danger [15,16]. Meanwhile, the singular cardiovascular disease case associated with SAH in our cohort suggests limited conclusions but aligns with known associations between vascular pathology and SAH presentation [16].

These results carry important clinical and public health implications. The high prevalence of hypertension and diabetes, both major modifiable risk factors, highlights the urgent need for robust prevention strategies, including community-level blood pressure screening, diabetes control programmes, and public education. Our results echo the Million Death Study results, which reported that hypertension was associated with an eight-fold increase in stroke mortality, and diabetes doubled stroke risk in Indian adults [17,18]. Aggressive risk factor management, through lifestyle changes and medication adherence, must be central to reducing the observed stroke burden.

In addition, the predominance of ICH in tertiary settings suggests that many ischaemic stroke cases, possibly less severe, may not reach referral centres, skewing hospital-based data. This bias aligns with the findings of the AHA registry in India, where younger patients and more severe strokes dominated tertiary admissions due to selection dynamics [16-18]. Key assets of this study include comprehensive imaging-based classification and a thorough risk factor assessment. However, limitations include a retrospective design, potential referral bias, the absence of a comparative ischaemic cohort, and a lack of long-term outcomes. The small number of SAH and EDH cases further constrains subtype-specific risk analyses. Future multi-centre prospective studies with community-level sampling and long-term follow-up are required to delineate risk-trait associations and outcomes in the broader Indian stroke population [18].

Our data reinforce that hypertension and diabetes are potent, underlying risk factors for haemorrhagic stroke in India. This study supports global and regional findings while contributing to local understandings of stroke subtype distribution. Talking about these modifiable factors through national health policy and structured clinical pathways is imperative to stem the tide of stroke-related illness and death.

This study has several limitations. Being retrospective and single-centred, selection bias cannot be excluded. The study lacked long-term outcome data and had a relatively small sample size for certain stroke subtypes. These factors may limit generalisability. Findings may apply to similar tertiary hospitals in North India but may not fully represent community-level stroke patterns.

## Conclusions

This study concluded that there is a predominance of ICH among hospitalised stroke patients in a tertiary care setting in Moradabad, with hypertension and diabetes mellitus emerging as the most significant and common modifiable risk factors. The majority of patients were elderly, and a moderate impairment in consciousness was observed at presentation. Significant associations were found between stroke subtypes and risk factors, mainly hypertension and diabetes, in haemorrhagic strokes. These results underscore the urgent need for region-specific methods focusing on initial detection and control of hypertension and diabetes, as well as public health involvement for stroke prevention. Assuming the disproportionately high rate of haemorrhagic stroke in this cohort, future community-based studies with larger sample sizes are necessary to validate these results and guide evidence-based healthcare planning in the region.
